# Best and worst performing health facilities: A positive deviance analysis of perceived drivers of primary care performance in Nepal

**DOI:** 10.1016/j.socscimed.2022.115251

**Published:** 2022-09

**Authors:** Todd P. Lewis, Amit Aryal, Suresh Mehata, Astha Thapa, Aisha K. Yousafzai, Margaret E. Kruk

**Affiliations:** a Department of Global Health and Population, Harvard T.H. Chan School of Public Health, 677 Huntington Avenue, Boston, MA, 02115, USA; b Federal Parliament of Nepal, Kathmandu, Nepal; c Ministry of Social Development, Biratnagar, Nepal

**Keywords:** Primary care, Quality of care, Nepal, Positive deviance, Qualitative methods

## Abstract

Primary care services are on average of low quality in Nepal. However, there is marked variation in performance of basic clinical and managerial functions between primary health care centers. The determinants of variation in primary care performance in low- and middle-income countries have been understudied relative to the prominence of primary care in national health plans. We used the positive deviance approach to identify best and worst performing primary health care centers in Nepal and investigated perceived drivers of best performance. We selected eight primary health care centers in Province 1, Nepal, using an index of basic clinical and operational activities to identify four best and four worst performing primary health care centers. We conducted semi-structured, in-depth interviews with managers and clinical staff from each of the eight primary health care centers for a total of 32 interviews. We identified the following factors that distinguished best from worst performers: 1) Managing the facility effectively, 2) engaging local leadership, 3) building active community accountability, 4) assessing and responding to facility performance, 5) developing sources of funding, 6) compensating staff fairly, 7) managing clinical staff performance, and 8) promoting uninterrupted availability of supplies and equipment. These findings can be used to inform quality improvement efforts and health system reforms in Nepal and other similarly under-resourced health systems.

## Highlights

• Local leaders and health workers felt good management was key to best performance.
• Best performers reported strong leadership at both the facility and local levels.
• Community accountability was also seen as a critical enabler of top performance.
• Leaders of worst performers were less responsive to facility and community needs.

## 1 Introduction

There has been renewed interest in primary care in recent years (Hone et al., 2018). When functioning optimally, primary care is an entry point to the health system and provides continuous, coordinated services to all people at an affordable cost (Starfield et al., 2005). These services will be essential to reaching universal health coverage and achieving the Sustainable Development Goals as reaffirmed in the 2018 Declaration of Astana (World Health Organization, 2018). In Nepal, a low-income country facing a growing double burden of infectious and non-communicable diseases, strengthening primary care performance is paramount (Gyawali et al., 2020).

In many settings, primary care services are poorly equipped to optimize health. Large-scale analyses show major deficits in the care people receive across countries and conditions, including in Nepal (Kruk et al., 2018). Recent nationally-representative analyses show low adherence to clinical guidelines for basic primary care services and poor performance of routine newborn care practices in Nepal (Kc et al., 2020; Lewis et al., 2019). Other studies highlight deficits in critical areas such as service readiness, staffing levels, and patient experience (Lama et al., 2020; Mehata et al., 2017).

Despite overall poor quality, data from direct observations of care in multiple low- and middle-income countries (LMICs) show large variations in primary care quality within countries (Kruk et al., 2017). This suggests that higher performance is attainable for some facilities, and that identifying and replicating practices used by best performing facilities may improve overall performance (Kruk et al., 2018). In Nepal, which began adopting a federal system of government in 2017, investigating performance variation is particularly timely. With federalization, there was a significant devolution of power from the federal level to seven provinces and 753 local governments. Municipal governments now play a direct role in the administration of primary health care centers and provision of services to local communities. This decentralization places high demands on local leaders in a health system with uneven distribution of expertise and resources (Vaidya et al., 2019).

Positive deviance analysis is an approach to quality improvement that identifies and learns from organizations and individuals who demonstrate exceptional performance (Bradley et al., 2009). Positive deviance can surface solutions to problems that use approaches and resources already available within a community, increasing the likelihood that new practices are adopted and sustained. The existing positive deviance literature largely focuses on specialized and hospital care in high-income settings (Austin et al., 2015; D'Aunno et al., 2018; Gabbay et al., 2013; Taylor et al., 2015; Toscos et al., 2018; Tsai et al., 2015).

This literature would be strengthened by extending inquiry to facility-level best practices at lower levels of care, especially in LMICs. Available positive deviance studies of primary care cite management capacity, support from local and district leaders, and community engagement as key factors differentiating best performance (Bradley et al., 2012; Fetene et al., 2016; Mabuchi et al., 2018). We build on this literature by applying the positive deviance approach to primary care in the under-resourced and newly-decentralized health system of Nepal. We investigate perceived performance drivers unique to this context and deepen understanding of performance drivers identified in previous research.

In this study, we sought to identify perceived drivers of primary care quality and explore how drivers generated good performance. We first developed a framework of potential drivers of facility performance to guide investigation. Using routinely collected health system data, we identified best and worst performing primary health centers in one province in Nepal and interviewed facility managers and clinicians to understand how perceived drivers influence facility performance. Evidence from this study can advance understanding of best-in-class facility performance drivers and inform quality improvement efforts in Nepal and elsewhere.

## 2 Methods

### 2.1 Study setting

This study took place in Province 1 in eastern Nepal. Nepal, a landlocked country in South Asia, has three distinct ecological zones: Terai (a lowland region), hills, and mountains. It has a population of over 29 million people and a gross domestic product (GDP) per capita of 1155 USD as of 2020. Life expectancy at birth is approximately 71 years. National health expenditure was 4.45% of GDP in 2019, with 0.8 physicians and 3.3 nurses and midwives per thousand people in 2019 (World Bank, 2019). Throughout the country, primary health care services are provided at the district level through health posts, primary health care centers (PHCCs), and district hospitals. Secondary and tertiary care are provided through regional hospitals and specialized facilities (World Health Organization Country Office for Nepal, 2007).

Our study was conducted in Province 1 which contains a variety of health settings, including both urban and rural areas, and a sufficient number of primary health care centers. Province 1 is composed of 14 districts and 137 municipalities, and all three of the nation's ecological zones. It contains approximately 40 public primary health care centers in addition to district hospitals, urban health centers, health posts, and several private facilities. Each PHCC has approximately three beds and should be staffed by one medical officer and at least eight additional health workers. Services include diagnosis and treatment of illness, basic services such as family planning and immunization, basic emergency obstetric and neonatal services, and laboratory services. PHCCs also oversee community-based services provided by mid-level health workers. Each PHCC is overseen by a local Management Committee composed of six to seven elected officials and local leaders. The 2019 New National Health Policy aimed to establish more advanced primary health care centers, known as primary hospitals, in each municipality; some primary health care centers had begun expanding staff and services towards becoming a primary hospital during this study. In 2021, when qualitative data collection took place, the COVID-19 pandemic was ongoing in Nepal.

### 2.2 Study design and conceptual framework

We conducted an in-depth qualitative study of eight primary health care centers in Province 1 to understand leader and clinician perspectives on the factors that distinguish best and worst primary care performance. We used a positive deviance framework to investigate why some PHCCs outperform others in this context (Bradley et al., 2009). We first used quantitative methods to identify best and worst performing PHCCs using routine health system data. We then used qualitative methods to develop rich insight into the factors that drive performance.

**Fig. 1:**
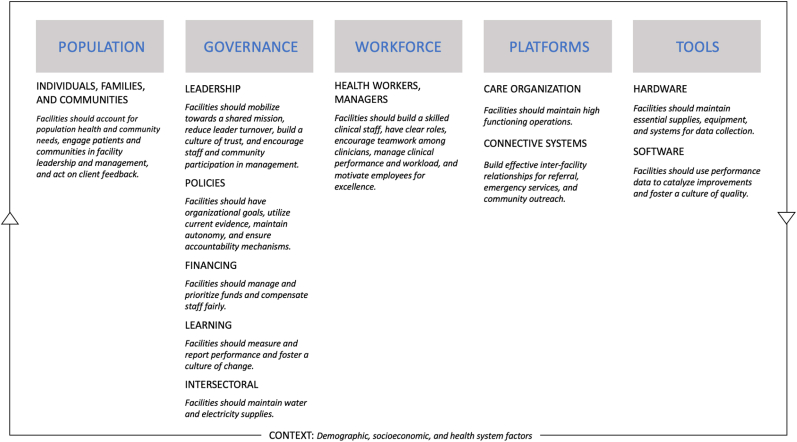
Hypothesized drivers of high performing health facilities.

To identify potential drivers of health facility performance for investigation, we reviewed organizational and management frameworks from multiple disciplines and mapped common factors to the foundations of high-quality health systems identified by *The Lancet Global Health Commission* on High Quality Health Systems in the Sustainable Development Goals Era (HQSS) (Fig. 1) (Kruk et al., 2018). The resulting conceptual model has five foundations composed of multiple domains: 1) Population (the role of individuals, families, and communities), 2) governance (leadership, policies, financing, learning, and intersectoral action), 3) workforce (management of the health workforce and its role), 4) platforms (health system care organization and connections), and 5) tools (hardware and software), and contextual factors (demographic, socioeconomic, and overall health system factors).

### 2.3 Identifying best and worst performers

We obtained 12 months of routinely-collected health system data at the facility level for all government-run PHCCs in Province 1 from April 2019 to March 2020 (approximately Baishakh 2076 to Chaitra, 2076 in the Nepali calendar), before the COVID-19 pandemic was widespread in Nepal. We reviewed available indicators and selected all those that were relevant to either clinical or operational performance based on the HQSS framework and discussions with local health system leaders, including the Chief of the Public Health Division in the Ministry of Social Development in Province 1 and his technical staff, on what constitutes good primary care performance in this setting. For clinical performance, we included: 1) the percent of children under five years with pneumonia who received antibiotics, 2) the percent of children under five years with diarrhea who were treated with zinc and oral rehydration salts (ORS), and 3) the percent of newborns who had chlorhexidine ointment applied immediately after birth. For operational performance, we included: 1) the percent of planned immunization clinics conducted out of a minimum of three per month required by national guidelines, 2) the percent of planned immunization sessions conducted out of a minimum of one per month required by national guidelines, and 3) the vaccine wastage rate across eight commonly offered vaccines as per the Nepal vaccine schedule. Other indicators in the health management information system (HMIS) measure utilization only, are not reported by primary health care centers, or were not consistently available for the time period of interest. Data are derived from facility self-report. Health workers at each PHCC are responsible for collecting data on a nationally-standardized set of indicators through facility registers and reporting to the municipal (palika) level where the data are audited. Data is recorded and stored in the national HMIS platform to which co-authors in the Ministry of Social Development had access. Subject matter experts in Province 1 reviewed the indicator set and verified the quality and completeness of data. A facility's performance score was calculated as the average of the six indicators. We ranked the performance scores for each facility from best to worst and identified the four best performing and four worst performing PHCCs. To assess robustness of results, we also calculated the standard deviation of a facility's performance score across the 12 months and found that the best performing facilities also had the lowest variability. Province 1 health system leaders reviewed selected facilities and agreed with the categorization of best and worst performers, providing face validity for the selection.

### 2.4 Study sample and data collection

Qualitative data were obtained through 32 semi-structured in-depth interviews conducted with four respondents at each of the eight PHCCs (Appendix table 1). We sought perspectives on facility performance from individuals performing diverse and critical roles, totaling four interviews per PHCC. We used a criterion sampling approach to identify each of the following respondents: one member of the Management Committee, the facility in-charge (typically a physician with both administrative and clinical duties), one advanced clinician (a physician or other senior clinician), and one nurse or auxiliary health worker. When possible, we sought interviewees who had been employed at the facility for at least two years, worked at the facility fulltime, and supervised or directly provided primary care services. We first contacted the facility in-charge of each PHCC to request participation; the in-charge helped to identify each additional interviewee from the facility according to the established criteria. The number of sites and respondents was selected to obtain a wide breadth of viewpoints and representation of multiple facility stakeholders; theoretical saturation within facility was typically achieved with fewer than four respondents (Rose and McCullough, 2017).

**Table 1:** Overview of best and worst performing primary health care centers in Province 1, Nepal.^a^

Best performers	PHCC-1	PHCC-2	PHCC-3	PHCC-4
Location	Semi-urban area in the hills	Rural area in the terai	Rural area in the terai	Rural area in the hills
Catchment population	5755	6275	6634	1660
Patient volumes	100-150 per day	∼200 per day	170-200 per day	10 per day
Total staff	16	17	14	9
24-h services	Yes	No	Yes	Yes
Municipal (palika) population	55,230	38,110	36,100	34,000
Health workers per 1000 people	1.4	1.7	1.7	1.5
Doctors and nurses per 1000 people	0.7	0.9	0.7	0.8
**Worst performers**	**PHCC-5**	**PHCC-6**	**PHCC-7**	**PHCC-8**
Location	Semi-urban area in the terai	Rural area in the hills	Rural area in the hills	Rural area in the mountains
Catchment population	9271	4018	4692	2616
Patient volumes	35-40 per day	30-35 per day	15-20 per day	∼45 per day
Total staff	12	15	10	11
24-h services	No	Yes	No	Yes
Municipal (palika) population	93,128	14,034	56,150	10,891
Health workers per 1000 people	1.0	2.5	1.1	2.0
Doctors and nurses per 1000 people	0.5	0.9	0.5	1.0

a Health workers and doctors and nurses per 1000 people are measured at the municipal (palika) level.

All interviews were conducted in Nepali from February to May of 2021 using a standardized interview guide tailored to each job category (Appendix exhibits 1–3). Interview guides were pre-tested within the research team and piloted with respondents at a PHCC in Province 1 that was not selected for the study. For the safety of the research team and respondents during the COVID-19 pandemic, interviews were conducted virtually and recorded using Zoom (Version 5.4.7, Zoom Video Communications Inc.). The research team ensured all participants had access to functioning internet and Zoom software; participant selection was not affected by access to these tools. Interview questions were based on the framework of potential performance drivers and covered topics such as facility management practices, the role of the community, and the role of local and municipal leaders. We asked questions such as “How do you set new rules or norms at this facility?” and “How is the relationship between facility managers and clinical staff?” Interviews were conducted by trained members of the research team with extensive knowledge of the Nepali health system and ranged from approximately 30 to 60 min. During the data collection period, the research team held meetings weekly or more frequently to debrief about findings, discuss emerging themes, and draft memos of initial perceptions. Three research assistants fluent in Nepali and English transcribed interviews verbatim and translated the interview recordings. To assure quality of the transcripts, bilingual research team members back translated sections of the transcripts and reviewed transcripts alongside interview recordings to verify accuracy and completeness. The Harvard T.H. Chan School of Public Health Institutional Review Board and the Nepal Health Research Council approved a verbal consent process and waived the requirement to document consent given the minimal risk to participants presented by this research.

### 2.5 Data analysis

To identify themes, we conducted a thematic analysis using both deductive and inductive approaches (Patton, 2015). Deductive codes were identified based on our performance framework. The research team reviewed the transcripts and interview memos to develop additional inductive codes. The preliminary codebook was applied to a subset of transcripts by two team members, after which codes were refined through research team consensus. This iterative process continued until no new concepts emerged and the final coding structure was obtained (Appendix table 2). We conducted an inter-rater reliability test for a subset of transcripts to ensure consistency between coders, with a Cohen's Kappa of 0.93. Two members of the research team then coded all remaining transcripts and interview memos.

**Table 2:**
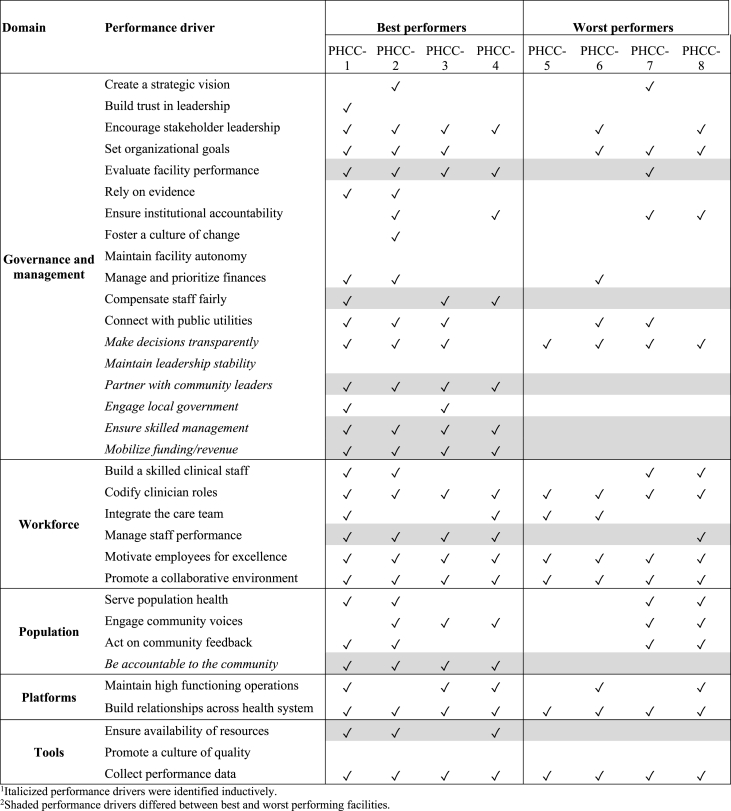
Perceived performance drivers in best and worst performing primary health care centers in Province 1, Nepal.

We used the constant comparative method for subsequent analysis in two phases. First, data were organized to provide a basic description of each PHCC and to identify factors that were perceived to promote or inhibit performance by multiple respondents at each facility. We also assessed consistency of responses among respondents and the importance ascribed to each factor to identify performance drivers perceived to be meaningful by respondents. Second, we compared the factors that were consistent across the majority of best performers and, separately, the majority of worst performers, to identify the factors that differentiated performance between the two groups. We sought out counter-examples of positive aspects in worst performers and negative aspects in best performers. Key themes were triangulated through similar analysis of interview memos. We used Dedoose (Version 8.3.47, SocioCultural Research Consultants, LLC, Los Angeles, CA, USA) to facilitate data coding, organization, retrieval, and visualization. Key results will be summarized, tailored to group, and shared with province, district, and municipal health system leaders.

### 2.6 Research team and reflexivity

Regular team debrief meetings were used to discuss emerging findings, which highlighted differences in researcher perspectives on how concepts apply within Province 1. Local team members noted important considerations for data collection practices, including the gender and ethnicity of the interviewers, and critical areas for exploration in interviews. Experts within the co-author team also vetted best and worst performers to enhance face validity. In team meetings, Nepali research team members helped to contextualize respondent perspectives within their experience as users and leaders of the local health system. The team also influenced the coding and analysis process, identifying key concepts in the transcripts that are particular to the Nepali context, such as the unique role of Management Committees. Overall, these differences in experiences and perspectives yielded a more thorough and balanced interpretation of the data.

### 2.7 Ethical approval

2.7

All research procedures were approved by the Harvard T.H. Chan School of Public Health Institutional Review Board and the Nepal Health Research Council.

## 3 Findings

Among all PHCCs in Province 1, performance scores ranged from 69% to 96% with a median score of 86%. Quality scores were 95% or above in the four highest ranked primary health care centers (best performers) and 75% or below among the lowest ranked (worst performers) (Appendix table 3; appendix Fig. 1). Table 1 describes the operational profile of each selected PHCC. The identified facilities are similar in staffing and services provided and span eight different districts across the three ecological regions of Nepal. In our qualitative analysis, we identified the deductive and inductive performance drivers perceived as meaningful by respondents (Table 2). Among these, eight key themes distinguished performance between best and worst performing PHCCs. Governance factors included: 1) Managing the facility effectively, 2) engaging local and municipal leadership, 3) developing sources of funding, 4) compensating staff fairly, and 5) assessing and responding to facility performance. We also identified one factor each among the domains for workforce, population, and tools: 5) managing clinical staff performance, 6) building active community accountability, and 7) promoting uninterrupted availability of supplies and equipment.

### 3.1 Managing the facility effectively

Best performing PHCCs reported high-quality management practices. Effective management was characterized by a range of practices from encouraging staff engagement to building a collaborative work environment between managers and staff. In contrast, worst performers described more disengaged staff and a weaker relationship between staff and facility leaders. Critical to this theme was a strong facility in-charge, regardless of clinical training, who catalyzed and maintained these practices within the facility. In particular, best performing facilities had regular team check-ins:

*We conduct staff meetings monthly … We discuss in these meetings who is doing what kind of work, any mistakes from any staff, which problem originated from where and how to solve them (Physician in-charge, PHCC-1, best performer).*

*Mostly we discuss queries, complaints, problems faced by staff regarding services, and what we should do to provide effective services and how we can provide better services … and if a problem should be represented to [higher levels of authority], then we will coordinate there too. So, these are the things we discuss regularly (Senior health assistant in-charge, PHCC-2, best performer).*

Worst performing PHCCs were less likely to engage their staff or involve them in important facility decision-making processes:

*There is no [monthly meeting with the in-charge] meeting to date. This is a huge gap. [If we had a regular meeting], I think things would get done. We have so many problems in the birthing center [at the PHC] (Auxiliary nurse midwife, PHCC-5, worst performer).*

Best performers described building an effective work environment by providing and accepting feedback and responding to staff concerns, as well as working together to jointly solve problems:

*As someone responsible for management, the team provides me feedback and talks to me about everything … We prioritize their queries and problems and do our best to solve them (Senior health assistant in-charge, PHCC-2, best performer).*

In contrast, worst performers did not feel the same degree of collaboration and responsiveness from management.

Finally, worst performing facilities lacked a collaborative culture. In contrast, facility managers in best performing PHCCs reported a strong institutional culture in which managers and staff had a positive working relationship:

*We are at this level and leading this PHCC because of the contributions of all staff. Not only because I am in management, but because of my supporters, all my friends, doctors, nurses, lab staff—the reason we are here is due to their joint and close relationships (Senior health assistant in-charge, PHCC-2, best performer).*

### 3.2 Engaging local leadership

Best performing PHCCs described strong relationships with their local Management Committee. They viewed the Management Committee as accessible, responsive, and integral to the facility's management processes and leadership team. While the staff of even the best performing facilities sought more engagement from their Management Committees in certain areas, respondents from best performers clearly described their Commitee as an important and effective advocate for the PHCC:

*It has been easy to work with the Management Committee. The chairman of the Management Committee listens to the matters of the health staff. He is very easy to approach and is like our guardian (Senior auxiliary nurse midwife, PHCC-1, best performer).*

*We feel secure with the management committee. They are the people’s representative. The chairman of the management committee is the ward chairman and they are local so we feel kind of secured … The ward chairman himself visits the municipality and talks to them about our PHCC. The Committee members are very helpful (Auxiliary nurse midwife, PHCC-2, best performer).*

Management Committees of best performing facilities also performed regular management functions and were seen as well-integrated facility leaders, often providing feedback or conducting monitoring visits at the facility. Committee visits were seen as helpful to ensuring high quality services for users and a good work environment for staff:

*We have regular meetings on a fortnightly or monthly basis. We ask the staff here if they have any problems or if they haven't received leave from the doctor … We tell them: "If you are facing any problems or issues like not receiving your salary, not getting leave, or not receiving over time compensation, let us know and we will make sure you receive them.” We will make demands to the respective authority on your behalf (Management Committee vice president, PHCC-1, best performer).*

Worst performing facilities described less reliable Management Committees who were often unavailable for meetings and unresponsive to facility needs. They typically had weaker or no monitoring functions and rarely visited the facility:

*There is exactly no relationship with them. I have been here for one and a half years and they have not visited the PHCC. During this crisis time of COVID-19, they also did not show any concern. They are responsible for managing our problems and visiting the PHCC, but there is no such thing. They do not visit even if we call them. Our relationship is not good (Auxiliary nurse midwife, PHCC-6, worst performer).*

Effective Management Committees also engaged external stakeholders for best performing PHCCs. In particular, they lobbied the municipality for funding for supplies and equipment and to expand services. Their ability to work with the municipality and mobilize funds were perceived to have helped best performing PHCCs avoid some of the resource constraints and management deficits keenly felt by worst performing PHCCs:

*If we have any problems, [the Management Committee] comes to support us, saying that we are beside you and we will fully support you. If anything deteriorates, we solve this in our Management Committee meeting. For example, the Committee has played a great role in initiating health insurance services here. We did not have a pharmacy; they constructed a pharmacy room here (Physician in-charge, PHCC-1, best performer).*

In comparison, Management Committees of worst performers were disengaged and less able to marshal support for the facility, even if funds were available from the municipality:

*The coordination between staff and management committee was not great. Before me, a doctor had come here with the aim to run emergency services. The municipality had also approved the budget. But the emergency services were not run because the Committee said that the staff would not receive money for extra duty. Because the coordination was not good, the approved budget was never used by the PHCC. As a result, no emergency services were provided, and the poor people had to suffer (Physician in-charge, PHCC-5, worst performer).*

### 3.3 Building active community accountability

Staff and leaders in the best performing facilities felt accountable to their local communities. They regularly solicited feedback from users and community members, typically in an informal fashion, and received additional feedback from the Management Committee. Best performers were seen as more likely to act on community feedback and find ways to involve the community in facility management:

*When I first came here, community members were raising concerns about the staff not being present in the PHCC in a timely manner. We held a meeting and sorted out this problem. (Health assistant in-charge, PHCC-3, best performer).*

*[The municipality and mayor] ask people about the quality of the services as well as any weak points in service delivery. They conduct board meetings every month and take feedback from the people during the meeting. They invite other politicians from other wards and they give us feedback received (Physician in-charge, PHCC-1, best performer).*

While worst performers reported receiving feedback from users, few had mechanisms for addressing issues or examples of remediating problems. Often, feedback mechanisms were ad hoc or addressed on an annual basis. In the few instances when problems were raised with the Management Committee or municipality, staff at worst performers felt it more difficult to find support or solutions:

*Truthfully speaking, there isn't substantial communication between [the clinical staff] and the community (Physician, PHCC-8, worst performer).*

### 3.4 Developing sources of funding

Best performing health facilities were able to access funds for their essential programs, medicines and supplies, and equipment, either through internal sources or through support from the Management Committee. For example, one facility described selling medicines to raise funds to buy other medicines they lacked. While no facility was free of funding shortages, the best performers described finding ways to cope and reinvesting funds in the facility:

*We do not face shortages of materials or problems with equipment at all. We have internal sources [of revenue] so even if the municipality does not do anything, the PHCC can manage (Physician, PHCC-2, best performer).*

Worst performers described ongoing funding shortages that hampered service provision. They often lacked the ability to mobilize funds from the Management Committee or the municipality. One PHCC was unable to access essential funds already allocated to their insurance program and Safe Motherhood Program because of the Management Committee's mismanagement of funds:

*I wanted to hold a meeting with the*M*anagement Committee in order to start the insurance program … The account is still in the names of a senior doctor who has already left this PHCC and a past president of the Management Committee. The account was not even transferred to the current Management Committee … The account is blocked because it is not in use (Physician, PHCC-6, worst performer).*

### 3.5 Assessing and responding to facility performance

Another differentiator between best and worst performing PHCCs was whether they tracked data on facility performance and used it to evaluate and improve their services. While no facility described a thorough target-setting procedures or indicators that capture processes or outcomes of care, best performers described regular recording and reporting procedures and engaged staff in these processes:

*I think tracking indicators helps us increase the quality of services. It helps us to know if we are working according to the targets or not. It also helps us to understand why people are not taking our services or if after our services they are satisfied or not. And it is helpful to solve any problems (Auxiliary health worker, PHCC-4, best performer).*

Target-setting was limited to measures of utilization and coverage in most facilities; few mentioned measurement of process or outcome measures. However, most worst performing PHCCs collected little data and recorded and reported data irregularly. When they did collect data, they rarely reported findings to the staff.

### 3.6 Compensating staff fairly

Best performing facilities ensured their staff received reasonable salaries, that salaries arrived on time, and that staff were compensated for overtime duties, such as additional night-shifts to cover 24-h services. In most cases, it was the responsibility of the Management Committee or the municipality leadership to provide and guarantee these funds:

*There has been a system for providing incentive allowances, such as giving 35% of lab revenue to motivate the staff. This has been a precedent set by the municipality—a good precedent—to motivate and incentivize the staff (Physician in-charge, PHCC-1, best performer).*

In contrast, worst performers noted low salaries, especially in remote locations, and a lack of compensation for additional working hours*.*

### 3.7 Managing clinical staff performance

Best performing PHCCs described ensuring that clinical staff had adequate skills and training so they could perform confidently in their roles. Some facilities also appointed section leaders to promote staff leadership and autonomy in practice:

*I feel capable and ready because the experience and training I have from all these years working in many different places have helped me a lot. Although I may not be perfect in everything, I am confident about doing what is expected of me in my job description. I am doing it all quite well (Staff nurse, PHCC-3, best performer).*

Best performing PHCCs also had staff-wide procedures for delineating roles and for ensuring that workloads were reasonable and fairly distributed:

*All staff of the PHCC attend the meeting. We discuss and then determine the role of all staff members. We have a 24-hour birthing service so we also discuss night duty (Auxiliary health worker, PHCC-3, best performer).*

The worst performing facilities described struggling to manage staff workloads and delineate clinical staff roles. Staff reported feeling underprepared for the duties expected of them within the facility.

### 3.8 Promoting uninterrupted availability of supplies and equipment

No facility had an adequate supply of medicines or supplies; most described issues obtaining and maintaining essential equipment such as X-ray machines. Many had received essential equipment via donation but could not access a technician to make repairs. Leaders and staff described how these issues hampered daily service provision. However, best performing PHCCs mobilized support through their Management Committee or found alternative ways to obtain the minimum required infrastructure. Worst performing PHCCs similarly struggled to access supplies and equipment. However, in contrast to best performers, their leaders and staff described no alternative approaches to obtaining these resources if they were not readily available from the municipality.

## 4 Discussion

We found several key factors that respondents felt distinguished performance among best performing and worst performing primary health care centers in Nepal. Governance and management factors included effective management by leaders within the facility, the engagement of the local Management Committee, facility performance assessment and response, developing sources of funding, and fair staff compensation. We also found that best performers described stronger clinical staff performance management, community accountability mechanisms, and the ability to access and maintain supplies and equipment in comparison to worst performing PHCCs. Respondents did not perceive basic facility and local characteristics as critical drivers of performance. Evidence from this study indicates that leaders in best performing facilities felt highly motivated for success. They were more effective advocates than leaders in worst performing facilities, leveraging relationships with community and municipal leaders to find solutions to common constraints.

Our findings re-enforce those found in comparable positive deviance studies focused on lower levels of the health system. In their study of primary health care units in rural Ethiopia, Bradley and colleagues identified some similar key themes differentiating best and worst performers including managerial problem solving capacity, relationship with the district health office, and community engagement (Bradley et al., 2012). Mabuchi and colleagues investigated high and low performance under a performance-based financing scheme among primary health care centers in Nigeria. They identified community engagement and support and performance and staff management as areas differentiating performance (Mabuchi et al., 2018). We similarly find that leadership capacity, including performance management, and community engagement were considered essential to best performance in the Nepali context. Our study broadens understanding of how good management distinguishes best from worst performance, identifying critical managerial skills around staffing, funding, and resourcing primary health care facilities.

Our findings suggest that health system quality improvement efforts may benefit from strengthening the leadership capacity of health facilities. In this study, respondents reported that better performance was obtained through soft skills such as relationship-building with staff and local leaders. Capacity-building interventions should support facility leaders in developing these abilities, including motivating teams, creating a shared vision, and promoting collaboration (Rowe et al., 2010). Best performers outperformed worst performers despite respondents reporting facing similar constraints such as stockouts, inoperable equipment, and remote locations. Further, nearly all facilities described difficulties working with municipal leaders underprepared for oversight of primary care provision, though best performers were able to extract the support they needed by developing and nurturing strong relationships with these leaders. These findings suggest that investment in management and leadership capacity at the lower levels of the health system may be important to elevating performance among struggling PHCCs.

Previous research has found that high-quality management, broadly, is associated with higher-quality primary care (Fetene et al., 2019; Mabuchi et al., 2018; Macarayan et al., 2019; Marchal et al., 2010). In our study, strong in-charges used a range of management strategies, from having regular meetings that include all staff members to establishing a collegial culture where the staff feel responsible for one another (Taylor et al., 2015). Facility leaders in best performers also engaged staff in leadership decisions within the facility, especially in terms of workload sharing and budgeting priorities (Marchal et al., 2010). While respondents in all facilities noted issues with certain managerial functions, such as ensuring stocks of medicines or filling vacant positions, leaders of best performing PHCCs were able to overcome barriers that leaders in worst performers could not (Mabuchi et al., 2018). Importantly, no facility in-charge reported leadership or management training, and strong management was not tied to level of clinical education. This suggests that struggling facilities may need innately capable, motivated leaders or intensive management coaching and supports to improve performance.

Best and worst performing PHCCs were also distinguished by the perceived engagement of their local Management Committees. Effective Management Committees provided support for internal management functions at the facility, conducting monitoring visits and problem-solving jointly with staff and facility leaders. Strong Management Committees were also critical to the other key distinguishing features of best performers. Together with facility in-charges, Committee members ensured sufficient financial resources to support facility operations, provided staff incentives or overtime pay, secured new equipment, or helped get it repaired. Critically, the Management Committee and in-charges of best performers noted robust performance management functions in comparison to worst performers (Mabuchi et al., 2018; Taylor et al., 2015). They monitored facility performance through data collection and target-setting, and managed clinical staff roles, responsibilities and workloads. Strong managers also engaged clinicians in making decisions and solving problems. Our findings align with previous work showing that staff performance management is critical for improving provider practice. Multifaceted approaches in particular, such as group problem solving with training, have been shown to have a large effect on performance (Rowe et al., 2018).

In addition, Management Committees were essential liaisons to the health coordinator and other leaders at the municipal government level. Previous positive deviance work in Ethiopia identified a strong relationship with the local health office as critical to success (Bradley et al., 2012). While no facility in our study described a completely positive relationship with the municipal authorities, best performers had Management Committees that could mobilize support from the municipality when necessary (Fetene et al., 2016). With the recent health system decentralization in Nepal, municipal governments assumed authority over local PHCCs' functions, such as procurement and staffing. While shifting power to local authorities has been documented to have positive effects, it must be accompanied by capacity building and accountability mechanisms for new leaders (Thapa et al., 2018). Municipalities currently lack clear roles and responsibilities, often receiving little guidance and few resources from higher levels of authority (Thapa et al., 2018; Vaidya et al., 2019). They may also lack the management skills, decision-making autonomy, and knowledge of procurement required to fulfill their duties. While best performers in our study were able to overcome some of these issues, structural changes are needed to prepare municipal governments to support struggling facilities, including strong financing, adequate human resources, and national quality standards (Bradley et al., 2011).

Our findings also demonstrate the importance of community accountability mechanisms in local PHCCs (Fetene et al., 2020). Managers and clinical staff in best performers reported feeling more accountable to their local communities than worst performers (Dieleman et al., 2009). They also reported acting on feedback from patient visits and community meetings in a timely manner, whereas worst performers often reported lacking the know-how or municipal support necessary to respond. Social accountability interventions in Nepal and other low- and middle-income countries have been shown to improve service quality for maternal health by improving health system responsiveness, increasing community ownership, and involving the community in decision-making processes (Nepal et al., 2020).

Our findings build on evidence suggesting that health facility committees can be effective stewards of facility performance. Studies show that health facility committees, formal groups with community representation and an explicit link to a health facility, may be able to improve the quality and coverage of care and some health outcomes (Björkman and Svensson, 2009; Lodenstein et al., 2017b; McCoy et al., 2012). However, as in this study, community accountability mechanisms are often individualized rather than systematic and are highly dependent on contextual factors such as the authority of local and facility leadership (Falisse and Ntakarutimana, 2020; George et al., 2015; Lodenstein et al., 2017a). The mechanisms by which community accountability is established and leveraged to improve performance is an area ripe for further research.

This study is designed to generate hypotheses about the practices that lead to good performance in primary care. While respondents may not be able to perceive all factors that drive performance, such as health system factors, they can perceive and report on their experience within the facility sphere of control. Recent work such as *The Lancet Global Health* Commission on Financing Primary Health Care points to larger structural factors that likely impact performance and are worthy of testing in future research (Hanson et al., 2022). Other such factors include provider payment mechanisms, availability and distribution of health workers, health provider education, facility leader training, and health system accountability models. While these structural levers fall outside the scope of this study, they are critical for developing best practices testable in future inquiry, including quantitative research. Developing a fuller understanding of these complex phenomena may also require application of sociological and political economy methodologies that can illuminate the organizational behaviors that breed problematic performance (Ramsey, 2022).

This study also demonstrates the usefulness of positive deviance analysis in understanding primary care performance. Future research may benefit from replication of this approach in other LMICs to identify common factors that promote or inhibit quality across contexts. Positive deviance could also be expanded to the level of countries or regions to explore higher-level factors that enable best-in-class performance. This study also demonstrates the successful application of the positive deviance approach using an online platform, an important contribution when in-person data collection is not practicable. During data collection, PHCCs were open and providing essential services despite the ongoing pandemic. Our inquiry focused on perceptions of how facilities arrived at their current state, regardless of any pandemic effects. However, facilities reported feeling effects of the pandemic in similar ways, noting that COVID-19 exacerbated existing barriers to better performance, such as maintaining meaningful engagement with community leaders who no longer visited the facility.

Our study is subject to several limitations. First, as with other positive deviance research, this study investigated relatively few primary health care centers and findings are specific to the context of the local setting, in this case Province 1; replicating this work with additional facilities in other regions of Nepal would improve transferability of findings. Second, this study could only capture respondents' perceptions of the factors that drive performance but is unable to test whether these factors had any significant effect or establish causal links with best or worst performance. Third, we were unable to blind all interviewers to the best or worst status of health facilities, which could bias how interviewers probed during interviews. Interviewers were trained to be even-handed in data collection and did not know any particular respondent's status or affiliation, and real-time debriefings during data collection may have mitigated bias. Previous work has shown that blinding in positive deviance analyses may be unnecessary (Rose and McCullough, 2017). Fourth, performance indicators for identifying best and worst performing facilities could be influenced by the number of patient visits. For example, facilities with higher volumes may have greater financial resources. Finally, this analysis could not account for contextual factors such as local poverty, patient volumes, or availability of health workers that might contribute to observed differences in facility performance. In particular, our methodology does not allow us to investigate factors affecting facility performance that originate upstream, such as preservice education, financing structures, or other macro-level determinants of health system performance; respondents may not be able to perceive these distal causes. However, respondents were able to tell a sequential story of perceived determinants of performance rather than merely describing features of best or worst performing facilities. These perceptions generate hypotheses that should motivate further investigation in qualitative studies and large quantitative studies, including at higher levels of the health system.

## 5 Conclusions

Our findings demonstrate the perceived importance of high-functioning leadership at the facility and local levels to achieving best clinical and operational performance in primary care facilities. In addition to its intrinsic value for facility operations, high-quality management may be able to improve access to resources, enhance performance assessment, and increase engagement with communities. An unexpected contribution of this work is the importance of good municipal leadership; the capacity of these leaders was considered as critical to performance as factors more proximal to facilities such as clinical skills or equipment.

Efforts to improve health system quality should invest in managerial and leadership capacity-building within facilities and local authorities that oversee health care provision. This is especially salient in the context of decentralization of health care delivery in Nepal. While additional research is needed, this is likely to be relevant in other decentralized contexts. Future work should develop best practices for strengthening local governance and generating community accountability mechanisms to improve primary care performance across under-resourced health systems.

## Author contributions statement

Todd P. Lewis: Conceptualization, Methodology, Formal analysis, Investigation, Data Curation, Writing—original draft, Project administration; Amit Aryal: Methodology, Formal analysis, Data Curation, Writing—reviewing and editing; Suresh Mehata: Methodology, Investigation, Writing—reviewing and editing; Astha Thapa: Investigation, Data Curation; Aisha K. Yousafzai: Methodology, Writing—reviewing and editing, Supervision; Margaret E. Kruk: Conceptualization, Methodology, Writing—reviewing and editing, Supervision.

## Data Availability

Data will be made available on request.
