# Supplementary material for: Best and worst performing health facilities: A positive deviance analysis of perceived drivers of primary care performance in Nepal
Source: Soc Sci Med. 2022 Sep;309:115251. doi: 10.1016/j.socscimed.2022.115251 (PMC9458868; doi:10.1016/j.socscimed.2022.115251)
Supplement: Multimedia component 1 [file mmc1.docx]

**Appendix**

**Appendix table 1: Respondents from best and worst performing primary health care centers in Province 1, Nepal**

|  | **Province 1 (14 districts with 137 municipalities)** | | | |
| --- | --- | --- | --- | --- |
| **Respondent type** | **PHCC-1: Best performer** | **PHCC-2: Best performer** | **PHCC-3: Best performer** | **PHCC-4: Best performer** |
| Management Committee member | Management Committee Vice President (3.5 years) | School principal (unknown) | School principal (9 years) | Ward chairperson (3 years) |
| Facility in-charge | Physician (1 year) | Senior health assistant (1.5 years) | Health assistant (2 years) | Health assistant (5 years) |
| Advanced clinician | Health assistant (1.5 years) | Physician (1.5 years) | Auxiliary health worker (28 years) | Auxiliary health worker (1.5 years) |
| Nurse or auxiliary health worker | Senior auxiliary nurse midwife (5 years) | Auxiliary nurse midwife (1.5 years) | Staff nurse (1.5 years) | Staff nurse (1 year) |
|  | **PHCC-5: Worst performer** | **PHCC-6: Worst performer** | **PHCC-7: Worst performer** | **PHCC-8: Worst performer** |
| Management Committee member | School principal (2 years) | School principal (20 years) | Ward chairperson (3.5 years) | Municipal health coordinator (14 years) |
| Facility in-charge | Physician (2 years) | Physician (6 months) | Health assistant (1 year) | Physician (2 years) |
| Advanced clinician | Senior auxiliary health worker (13 years) | Auxiliary health worker (2.5 years) | Physician (6 months) | Physician (2 years) |
| Nurse or auxiliary health worker | Auxiliary nurse midwife (1 year) | Auxiliary nurse midwife (2 years) | Auxiliary health worker (1.5 years) | Staff nurse (2 years) |

**Appendix exhibit 1: Qualitative interview guide for facility leaders**

**Positive deviance analysis of care quality in Nepal**

Semi-structured interview protocol—Facility in-charge

*[Please note: The interview will be a conversation and the following should be considered a general outline.]*

PREAMBLE: Thank you so much for offering me your time today. Is now still a good time to speak for the next 45 to 60 minutes? My name is [INTERVIEWER NAME]. I am working in conjunction with the Ministry of Health and a team of researchers on a study of the health system here in Province 1. The purpose of this interview is to understand more about how primary health care centers operate in the region. You are not being evaluated and there are absolutely no right or wrong answers to any of these questions; I am simply interested in hearing any insight or experiences you would like to share as a manager of this facility. I want to hear your honest opinions, what is working well, and how the health system could work better.

Typically, we record these interviews to make sure we don’t miss anything you say, but **I will never share anything you say in connection with your name. We will only save the sound recording, not the video, and we will never share your name or your PHC.**

- Do I have your permission to record?
- Do you have any questions?

Please feel free to ask questions or stop the interview at any point. To begin, I would like to ask you a few questions about this facility and your role.

**Background information**

|  | **Facility operations** |  |
| --- | --- | --- |
| 1 | What is the catchment area of this facility? |  |
| 2 | What services do you offer at this facility? |  |
| 3 | How many employees work at this facility in total? |  |
| 4 | How many physicians work regularly at this facility? |  |
| 5 | How many nurses work regularly at this facility? |  |
| 6 | What facility is your nearest referral center? |  |
| 7 | What sources of funds does your facility have? |  |
|  |  |  |
|  | **Managerial role** |  |
| 8 | What is your position? |  |
| 9 | What is your level? |  |
| 10 | What was your training? |  |
| 11 | How long have you been working in this facility? |  |
| 12 | How long have you been in a management role? |  |
| 13 | Have you had any formal training as a manager?  [If yes] What sort of training did you receive? |  |
|  |  |  |
|  | **Community and patients** |  |
| 14 | How many patients does this community serve in one week? |  |
| 15 | What are the three most common reasons for individuals to visit this facility? |  |

**Facility team and culture**

1. **Please describe the clinical staff at your facility. How do you feel they perform?**
   1. What criteria do you use to assess performance?
   2. How do you provide feedback to clinicians?
2. **How is the relationship between facility managers and clinical staff?**
3. How do you build trust among employees?
4. How do you support employees at this facility, if at all?
5. **How do you determine clinical roles at your facility (who does what)?**
6. What sort of tools/guidelines do you use to determine responsibilities?
7. How do you ensure clinicians fulfill their roles?
8. **How do you get clinicians in your facility to work together?**
9. What promotes teamwork within your facility?
10. What inhibits teamwork within your facility?
11. **How do you maintain motivation among clinicians?**
12. What encourages good care at your facility?
13. What inhibits good care at your facility?
14. **How do you solicit feedback from employees, if at all?**
15. How do you implement changes based on this feedback?
16. How do you ensure clinicians feel free to share opinions with managers?

**Management**

1. **How do you set new rules or norms for your facility?**
   1. How do you disseminate new rules/norms among employees?
   2. How do you build support for new rules/norms, if at all?
2. **How do you involve other staff members in management decisions, if at all?**
3. **What is the role of local level political leadership in managing this facility?**
4. How does local leadership promote good performance at this facility?
5. How does district leadership inhibit good performance at this facility?
6. **What is the role of district and province level leadership in managing this facility?**
   1. How does district/province leadership promote good performance at this facility?
   2. How does district/province leadership inhibit good performance at this facility?
7. **How does your facility communicate with other facilities, such as to obtain advice about patient care?**
   1. How frequently is your facility in touch with other facilities?
   2. How does your facility use information from other facilities, if at all?
8. **What relationship does your facility have with other facilities in the area, if any?**
   1. How do other facilities support your facility, such as through peer support or learning collaboratives?
9. **What is your role in procuring medicines and supplies for this facility, if any?**
   1. Can you quickly get the medicines and supplies you need?
   2. How do you influence this process?
10. **What is your role in buying or repairing equipment for this facility, if any?**
    1. Can you quickly get the equipment you need?
    2. How do you influence this process?
11. **What is your role in selecting who works at your facility, if any?**
    1. How are you involved in hiring and firing employees?
    2. How do you influence the types of staff members you employ?

**Accountability**

1. **What sort of goal-setting do you do, if any, and what is that process like?**
   1. How did you develop these goals?
   2. How do you hold yourself accountable to goals, if at all?
   3. How do you hold employees accountable to goals, if at all?
2. **Who are you accountable to for performance of this facility, if anyone?**
3. How do district and province leaders hold this facility accountable?
4. How does the community hold this facility accountable?
5. **How do you understand “quality of care” at the point of care?**
   1. What does “quality of care” mean in this facility?
6. **How do you monitor quality of care in your facility?**
7. What sort of metrics do you use, if any?
8. How did you choose these metrics?
9. How do data systems support your work? What could be improved?

**Relationship with the community**

1. **How does the local community influence facility performance, if at all?**
   1. How important is the local community to facility functions, if at all?
2. **How do you assess the health needs of your local community, if at all?**
3. What helps you meet the needs of the community?
4. What makes it difficult to meet the needs of the community?
5. **How do you share information with the community?**
6. How do you build transparency on facility performance, such as finances?
7. **How do you obtain feedback from patients and community members, if at all?**
8. Please describe this feedback.
9. **What actions have you taken in response to community feedback, if any?**

**Overall perception of facility**

1. **Overall, what do you think of the quality of care provided by this facility?**
   1. Why do you think the quality is good/poor?
2. **What are the most important factors to providing high quality care?**
3. **What are some successes you have had in managing this facility?**
   1. What did you learn from these successes?
4. **What are the primary challenges you have faced managing this facility?**
5. What did you learn from these challenges?
6. **What would it take to make this the best performing health facility in this district?**

CONCLUSION: I notice we are nearing the end of our time today. **Before we close, is there anything we have not asked you that you think is important to tell us regarding the performance of your facility?** Is there anything more you would like to ask me?

*[Identifying other interviewees]*

Before we end, I would like your help identifying two clinicians at your facility who would be able to speak with us about their role. We are interested in speaking with a physician and a nurse who regularly see patients and have worked at this facility for a few years.

We would also like to speak with a community representative on the facility board.

Could you provide us with the names and contact information of these individuals?

I want to sincerely thank you for your time today. Please contact me with any questions. Have a great day!

**Appendix exhibit 2: Qualitative interview guide for Management Committee members**

**Positive deviance analysis of care quality in Nepal**

Semi-structured interview protocol—Facility Management Committee member

*[Please note: The interview will be a conversation and the following should be considered a general outline.]*

PREAMBLE: Thank you so much for offering me your time today. Is now still a good time to speak for the next 45 to 60 minutes? My name is [INTERVIEWER NAME]. I am working in conjunction with the Ministry of Health and a team of researchers on a study of the health system here in Province 1. The purpose of this interview is to understand more about how primary health care centers operate in the region. You are not being evaluated and there are absolutely no right or wrong answers to any of these questions; I am simply interested in hearing any insight or experiences you would like to share about this facility. I want to hear your honest opinions, what is working well, and how the health system could work better.

Typically, we record these interviews to make sure we don’t miss anything you say, but **I will never share anything you say in connection with your name. We will only save the sound recording, not the video, and we will never share your name or your PHC.**

- Do I have your permission to record?
- Do you have any questions?

Please feel free to ask questions or stop the interview at any point.

**Warm-up**

1. **To get started, I would love to hear a bit about you. Please tell me a little about yourself.**
   1. How long have you been a board member at this facility?
   2. How would you describe your role and responsibilities?

**Facility team and culture**

1. **How do you feel the clinical staff at this facility performs?**
   1. What criteria do you use to assess performance?
2. **How is the relationship between facility managers and clinical staff?**
3. How do you support the manager at this facility, if at all?
4. How do you support employees at this facility, if at all?
5. **How do you solicit feedback from employees, if at all?**
6. How do you implement changes based on this feedback?
7. How do you ensure employees feel free to share opinions?

**Management**

1. **How do you set new rules or norms for your facility?**
   1. How do you disseminate new rules/norms among employees?
   2. How do you build support for new rules/norms, if at all?
2. **What is the role of local level political leadership in managing this facility?**
3. How does local leadership promote good performance at this facility?
4. How does district leadership inhibit good performance at this facility?
5. **What is the role of district and province level leadership in managing this facility?**
   1. How does district/province leadership promote good performance at this facility?
   2. How does district/province leadership inhibit good performance at this facility?

**Accountability**

1. **What sort of goal-setting do you do, if any, and what is that process like?**
   1. How did you develop these goals?
   2. How do you hold yourself accountable to goals, if at all?
   3. How do you hold employees accountable to goals, if at all?
2. **Who are you accountable to for performance of this facility, if anyone?**
3. How do district and province leaders hold this facility accountable?
4. How does the community hold this facility accountable?
5. **How do you understand “quality of care” at the point of care?**
   1. What does “quality of care” mean in this facility?
6. **How do you monitor quality of care in your facility?**
7. What sort of metrics do you use, if any?
8. How did you choose these metrics?
9. How do data systems support your work? What could be improved?

**Relationship with the community**

1. **How does the local community influence facility performance, if at all?**
   1. How important is the local community to facility functions, if at all?
2. **How do you assess the health needs of your local community, if at all?**
3. What helps you meet the needs of the community?
4. What makes it difficult to meet the needs of the community?
5. **How do you share information with the community?**
6. How do you build transparency on facility performance, such as finances?
7. **How do you obtain feedback from patients and community members, if at all?**
8. Please describe this feedback.
9. **What actions have you taken in response to community feedback, if any?**

**Overall perception of facility**

1. **Overall, what do you think of the quality of care provided by this facility?**
   1. Why do you think the quality is good/poor?
2. **What are the most important factors to providing high quality care?**
3. **What would it take to make this the best performing health facility in this district?**

CONCLUSION: I notice we are nearing the end of our time today. **Before we close, is there anything we have not asked you that you think is important to tell us regarding the performance of your facility?** Is there anything more you would like to ask me?

I want to sincerely thank you for your time today. Please contact me with any questions. Have a great day!

**Appendix exhibit 3: Qualitative interview guide for clinicians**

**Positive deviance analysis of care quality in Nepal**

Semi-structured interview protocol—Clinicians

*[Please note: The interview will be a conversation and the following should be considered a general outline.]*

PREAMBLE: Thank you so much for offering me your time today. Is now still a good time to speak for the next 45 to 60 minutes? My name is [INTERVIEWER NAME]. I am working in conjunction with the Ministry of Health and a team of researchers on a study of the health system here in Province 1. The purpose of this interview is to understand more about how primary health care centers operate in the region. You are not being evaluated and there are absolutely no right or wrong answers to any of these questions; I am simply interested in hearing any insight or experiences you would like to share as a health care worker at this facility. I want to hear your honest opinions, what is working well, and how the health system could work better.

Typically, we record these interviews to make sure we don’t miss anything you say, **but I will never share anything you say in connection with your name. We will only save the sound recording, not the video, and we will never share your name or your PHC.**

- Do I have your permission to record?
- Do you have any questions?

Please feel free to ask questions or stop the interview at any point.

**Warm-up**

1. **To get started, I would love to hear a bit about you. Please tell me a little about yourself.**
   1. How long have you been practicing at this facility?
   2. How would you describe your role and responsibilities?

**Facility team and culture**

1. **How do you determine your role and responsibilities as a clinician at the facility?**
   1. How do you know what your responsibilities are?
   2. How well do you think responsibilities are distributed across the clinical staff?
2. **How well do clinicians in your facility work together?**
3. What promotes teamwork within your facility?
4. What inhibits teamwork within your facility?
5. **How is your relationship with the managers of this facility?**
   1. How do managers build trust among employees?
   2. How do managers support clinicians at this facility, if at all?
6. **What motivates you to provide good quality care?**
7. What encourages good care at your facility?
8. What inhibits good care at your facility?
9. **How prepared do you feel to perform your duties?**
   1. How do you receive feedback from managers or others at the facility?

**Management**

1. **Please describe the management of the facility. How well are managers serving the needs of the facility?**
2. How well are managers serving the needs of the clinicians and staff?
3. How well are managers serving the needs of the patients?
4. **How do you feel this facility performs in terms of day-to-day operations?**
   1. What at the facility runs very smoothly? Why?
   2. What at the facility does not run smoothly? Why?
5. **How do managers solicit feedback from employees, if at all?**
6. How do you give feedback to managers within this facility?
7. How do managers implement changes based on this feedback?
8. **How are new rules or norms set for your facility?**
   1. How are these disseminated among employees?
9. **How are clinicians involved in management decisions at the facility, if at all?**
10. How much autonomy do you have to make decisions within the facility?
11. **How does your facility communicate with other facilities, such as to obtain advice about patient care?**
    1. How frequently is your facility in touch with other facilities?
    2. How does your facility use information from other facilities, if at all?
12. **What relationship does your facility have with other facilities in the area, if any?**
13. How do other facilities support your facility, such as through peer support or learning collaboratives?

**Accountability**

1. **What sort of goal-setting happens at the facility, if any, and what is that process like?**
   1. How do you hold yourself accountable to goals, if at all?
   2. How do managers hold you accountable to goals, if at all?
2. **Who are clinicians accountable to for their quality of care, if anyone?**
3. How do managers hold clinicians accountable?
4. How does the community hold clinicians accountable?
5. **When you hear the phrase “quality of care,” what does that mean to you?**
   1. What does “quality of care” mean in this facility?
6. **How is quality of care monitored in your facility?**
7. What sort of metrics do you use, if any?
8. How did you choose these metrics?
9. How do data systems support your work? What could be improved?
10. **How do you use evidence to improve the care you provide at this facility? Could you give me an example?**
11. What helps you make these changes in practice?
12. What makes it difficult to change in response to new evidence?

**Relationship with the community**

1. **How does the local community influence facility performance, if at all?**
   1. How important is the local community to facility functions, if at all?
2. **How do you assess the health needs of your local community, if at all?**
3. What helps you meet the needs of the community?
4. What makes it difficult to meet the needs of the community?
5. **How do you obtain feedback from patients and community members, if at all?**
6. Please describe this feedback.
7. **What actions have you or colleagues taken in response to community feedback, if any?**

**Overall perception of facility**

1. **Overall, what do you think of the quality of care provided by this facility?**
   1. Why do you think the quality is good/poor?
2. **What are the most important factors to providing high quality care?**
3. **What are some successes you have had practicing in this facility?**
   1. What did you learn from these successes?
4. **What are the primary challenges you have faced practicing in this facility?**
5. What did you learn from these challenges?
6. **What would it take to make this the best performing health facility in this district?**

CONCLUSION: I notice we are nearing the end of our time today. **Before we close, is there anything we have not asked you that you think is important to tell us regarding the work you do at your facility?** **Is there anything more you would like to ask me?**

I want to sincerely thank you for your time today. Please contact me with any questions. Have a great day!

**Appendix table 2: Codebook for analysis of best and worst performing primary health care centers in Province 1, Nepal**

| Domain | Code | Definition | When to use | When not to use | Example |
| --- | --- | --- | --- | --- | --- |
| Governance | Access to funds | Any comment regarding facility access to funds or availability of facility funding, positive or negative | This refers to how funds are raised/collected. | This does not refer to using or budgeting funds. | Most of our services are free in our health center since it is under government. We get funds from the municipality for PHC work provision and another one from the ward office where we ourselves go and visit the ward president in a meeting. |
| Population | Addressing community needs | Any comment on facility addressing population health and patient needs (i.e., local priority setting, monitoring common conditions, accounting for new disease outbreaks), positive or negative |  |  | When people could not cross the river during the rainy season and people were dying, we took the initiative to bring the army doctors to provide treatment. |
| Platforms | Basic operations | Any comment regarding management and operations (e.g., open at reasonable times, has health workers present 24/7, displays user fees), positive or negative |  |  | At first we were not able to provide a complete, basic level of services, but now, due to a large well-equipped building we are able to provide multiple services. |
| Context | Care organization | Any comment regarding how health system organization/care structures/care levels influence facility functions, positive or negative |  |  | Another thing is that many PHCs have been upgraded from health posts. So even though they are declared to be PHCs, their structure is that of health posts. Because of that, it is difficult to work. |
| Workforce | Care team integration | Any comment about integration of all clinicians into care processes (e.g., empowered nursing staff, integrated pharmacists), positive or negative | This refers to integrating team members in care provision. | This does not refer to integrating clinicians into administration/leadership. | But whenever there is any type of risk, we consult our seniors and discuss with our in-charge. I do not have SBA (skill birth attendant) training so I consult the SBA trained senior sister and then handle that case or refer it. |
| Tools | Clinical information/evidence | Any comment on obtaining/disseminating up-to-date information to clinicians, positive or negative | This refers to information accessed by or provided to staff. | This does not refer to knowledge of clinical practice. | I had a gap year after my studies, after which I went to a health post in [location]. The patient flow was not high in the health post. But when I went to the PHC, maybe because I got SBA immediately, I feel like I have gained a lot of knowledge. I have had the opportunity to observe many things. I have received training and also guidelines from doctors. It's good. |
| Workforce | Clinical staff skills | Any comment regarding recruitment/retention of competent, engaged, and adaptable health workers; overall quality of clinical staff performance, positive or negative | This refers to skills or knowledge about practice. | This does not refer to guidelines or trainings. | We have skilled staff here and they have provided services to people in the best way possible. |
| Workforce | Clinician roles | Any comment about clear, appropriate roles and responsibilities for facility staff, positive or negative |  |  | We hold meetings and have in a written format that a particular person will be responsible for a particular section. |
| Population | Community feedback | Any comment on soliciting client feedback, sharing feedback with the community, and taking appropriate action, positive or negative |  |  | Sometimes we receive good responses and sometimes bad. Some local people think that the PHC services should be available in the community which are not possible, like emergency services for people. Some have given good responses after receiving satisfying services. |
| Population | Community involvement | Any mention of engaging patients and communities in facility leadership and management (e.g., by having a community advisory board or a community member attend staff meetings) and account for community culture, traditions, preferences, and knowledge, positive or negative |  | This does not apply to facility staff engaged in leadership, even if they are part of the community. | We do have any program that involves directly working in the community. But we have chart boards in different places on topics of health, sanitation, and balanced diet. |
| Governance | Culture of change | Any comment regarding receptivity to change and growth, positive or negative | This refers to acceptance of and willingness to change. | This does not refer to quality improvement initiatives. | We all continuously discuss in our staff meeting how to strengthen and improve management of our PHC to provide more effective service and how to include client satisfaction with our service. |
| Tools | Culture of quality | Any mention of facilities determining, communicating, or fostering a culture of quality (mission, motivation, spirit, emphasis), positive or negative | This refers to institutional culture in terms of quality improvement, maintenance, or mission. | This does not refer to adapting or learning functions. | Personally, I'm happy when I'm able to deliver more than the available resources seem to allow. I feel like we've given many services here. |
| Governance | Decision-making | Any comment regarding how decisions are made at the facility and who is involved, positive or negative | This refers to decision-making at the facility level (among staff and managers) or among the Management Committee or upper-level government. |  | All decisions are made through the management committee meetings. |
| Context | Demographics | Any mention of community and catchment area demographics, such as urbanicity, volumes |  |  | From this ward, a total of 6,200 come for services. People come from another ward too but I do not know their exact population. |
| Governance | District/province/federal effectiveness | Any mention of how well the facility is able to work with upper level government officials at the district province or federal levels, how embedded they are, whether facility has access to power/influence, positive or negative |  |  | We remain in contact with the district only on issues related to immunizations, etc. But other than that, we do not even receive direct letters from the district. |
| Governance | District/province/federal role | Any mention of the role and responsibilities of various levels of higher government in relationship to the facility |  |  | There is not any direct program from the province. The province and health office are correlated and we coordinate with the health office for reporting. There is no such program from the province. |
| Tools | Essential resources | Any mention of the supply of drugs, supplies, and information systems, and ability to mobilize resources when necessary, positive or negative |  |  | We have experienced that the medicines that are sent in bulk to us are not the ones that we need the most. But the medicines we need on a regular basis are provided only on a nominal basis. |
| Governance | Facility autonomy | Any mention of aligning with the health system and/or maintaining autonomy as a facility, positive or negative | This should be used for autonomy is mentioned by clinical staff or management. We should look at them as one unit. |  | The economic status is also not good. The institution is dependent on others, so it is difficult to run this institution. |
| Context | Facility info | Any comment on general facility information, like opening times, location, geography, history, etc. |  |  | Other services are not available here. For example, people come for OPD services. We also do normal delivery. We haven't been able to provide more advanced services. |
| Governance | Facility management activities | Any mention of fundamental management activities (e.g., meetings, meeting minutes, reporting back to staff), positive or negative |  |  | Every month, we have a staff meeting in our PHC but, the committee meeting, our in-charge attends it and I don’t know other participants in the meeting. |
| Governance | Financial management | Any mention of facilities recording, managing, or prioritizing expenses, positive or negative | This refers to use, application, or budgeting of funds. | This does not refer to accessing or collecting funds. | From those earnings we do management like repairing offices, buying some small instruments. |
| Platforms | Health system relations | Any mention of developing or managing networks and relationships, such as emergency services, referral systems, and community outreach, positive or negative |  |  | We have good coordination. Sometimes we meet in training with all health staff. We have good coordination. We borrow medicines from other facilities when in need. |
| Tools | Infrastructure | Any mention of facility physical assets/infrastructure, positive or negative; any comment on adding or updating infrastructure (e.g., new buildings, new lab, new facility living quarters) | This refers to the building and structures on the facility premises. | This does not refer to basic utilities. | If road infrastructure were easier then it would also help with referral. We sent a patient and in the middle they had to change to another vehicle. |
| Governance | Institutional accountability | Any comment regarding internal and external accountability mechanisms for institutional change, positive or negative |  |  | We are first accountable to the citizens and then to the municipality. Most importantly, we are accountable to those who receive services at the PHC. |
| Context | Insurance mechanisms | Any mention of how insurance mechanisms influence facility performance, positive or negative |  |  | If people tend to get checked and treated in time then it won't create difficulties later. This has been promoted by the health insurance program to a greater extent. |
| Governance | Leadership experience/skills | Any mention of experience/training/qualifications of facility leadership, positive or negative | This refers to facility leadership preparedness. | This does not refer to other leaders, like managing committee members of palika officials. | I haven't received any administration or management related training. I've been learning by doing here. |
| Governance | Leadership stability | Any comment regarding turn-over of medical officer and/or managing committee, consistency of leadership, positive or negative |  |  | I am neither permanent nor contract-based. I was recruited by the Municipal Hospital. |
| Governance | Management committee effectiveness | Any mention of whether the management committee is effective or ineffective, and general qualities of the managing committee | This refers to how well the Management Committee executes its role. |  | They help with financial as well as manpower resources however they can. We have an internal fund too but if it’s not sufficient then they help in raising funds from the municipality and INGOs. |
| Governance | Management committee role | Any comment regarding the responsibilities and/or role of the managing committee or their relationship with the facility, positive or negative | This refers to the responsibilities expected of the Committee. |  | Management committee is the committee to resolve the problems faced by the PHC. We have a similar committee in schools. If a problem occurs while doing some major work, the committee meets and discusses the way forward. |
| Governance | Municipality effectiveness | Any mention of whether the municipality/palika is effective or ineffective, and general qualities of the municipal leadership | This refers to how well the municipality executes its role. |  | I do not know why this problem is arising. Local government should me making things more convenient. It might be because this area is very remote. |
| Governance | Municipality role | Any comment regarding the responsibilities and/or role of the municipality/palika or their relationship with the facility, positive or negative | This refers to the responsibilities of the municipality. |  | For example, we make the emergency services routine. But it is the local government who determines and provides the salary. |
| Governance | Organizational goals | Any comment regarding facility targets with specified timeframes supported by coordinated activities, positive or negative | This refers to specific goals set out by the facility, mostly in the short-term. | This does not refer to the facility's mission or long-term vision. | Our main goal now is to resume the insurance program run by the Nepal insurance board. |
| Governance | Performance tracking | Any mention of collection or use of performance data (e.g., tracking adverse events), monitoring progress, and transparency of results, positive or negative | This is for any use, reporting, or sharing of data. | This is not for guidelines or clinical evidence. | We have to send a monthly report to the municipality and that report will help with the annual report. It helps us to know the number of services we achieved. |
| Context | Political commitment | Any mention of relevant politicians' commitment to facility performance, positive or negative |  |  | We, all staff, are responsible for our work. Only a few aren’t since this area has a political impact and they are involved in it. |
| Governance | Public utilities | Any mention of linkage to basic resources/utilities like water and electricity, positive or negative |  |  | Our electricity is in yellow but everything else is in green. We did not have an electricity backup. But now a generator is being managed. |
| Context | Socioeconomic factors | Any mention of community socioeconomic factors such as education level, income, employment |  |  | And another is the transportation problem. We tell the patients that the case is serious and they have to go but they say that they do not have money and will not go. This creates trouble for us. If they do not go, the case will deteriorate. And we'll have to wait on them all the time. |
| Workforce | Staff appointment | Any mention of reason/motivation for staff appointment or placement, process for hiring at particular facility, positive or negative | This is for any comments about how staff are appointed, filling vacant roles, recruiting, etc. | This does not refer to turnover. | Not just in PHC, in health posts as well, advertisements are announced as soon as the position becomes vacant. |
| Governance | Staff compensation | Any mention of compensation for health workers, positive or negative | This could include incentives or bonuses. |  | Yes, there is an incentive to only those staff who perform night duty. The financial incentive is less, Nrs. 8000 per month and is divided per duty. |
| Workforce | Staff motivation | Any mention of incentivizing or motivating health worker engagement with rewards, incentives, and opportunities for promotion |  |  | This is also like god’s blessings. I feel somewhere this sense of responsibility because our profession is involved in the health of people. That’s why that responsibility, a sense of service, to implement something utilizing our knowledge and learning, these kinds of things motivate me to provide quality services. |
| Workforce | Staff performance management | Any mention of support for health workers through trainings, supportive supervision, and workload management, positive or negative |  |  | The staff working in OPD and dispensary say that the work load is too much. Because the patient flow is very high. They complain about a lack of enough manpower. |
| Governance | Stakeholder leadership (within facility) | Any mention of leaders engaging clinical voices at all levels, such as physician and nurse champions, positive or negative | This refers to involvement of staff members in facility administrative matters. | Does not apply to community engagement in leadership | We have such a type of staff that if there is any problem being faced, we resolve it together. |
| Governance | Strategic vision | Any mention of leaders establishing and communicating a shared mission (e.g., values, goals, strategy) or mobilizing key stakeholders for change, positive or negative | This refers to long-term vision and planning. | This does not refer to short-term targets or goals. | You asked about the long-term goal which is simply to provide inpatient available services, such as USG, snake bite management, delivery services. We want to add anesthesia, caesarian section services here. Soon, in about 2 years. we will be able to attain it. is my thinking. Until I am here, this will be my motto. |
| Workforce | Teamwork | Any comment regarding having a collaborative workplace culture with good communication, effective teamwork, and a strong peer network, positive or negative |  |  | For teamwork, like if there is training then we take turns so that each staff gets an equal chance. We share both benefits and work equally, that’s why everyone helps each other. |
| Governance | Trust in leadership | Any comment on leadership building a culture of trust within the organization or with external stakeholders, positive or negative |  |  | There is a big role. The major role is their trust in me and my institution. They have regained the feeling of trust in us. They are motivated to utilize services from me and my staff. |
| Governance | Understanding of quality (leadership) | Any mention of whether leadership has a meaningful, thoughtful, and appropriate understanding of quality of care, positive or negative; also for perception of the facility's quality | This refers to medical officers and management committee members. | This does not refer to clinicians and staff. | To us, quality health services means that all the health services that the Nepal government has included as basic rights is provided to the citizens in a convenient and timely manner by skilled health professionals. |
| Workforce | Understanding of quality (staff) | Any mention of whether staff has a meaningful, thoughtful, and appropriate understanding of quality of care, positive or negative; also for perception of the facility's quality, like "we have effective services" | This refers to clinicians and staff. | This is not for the facility medical officer or management committee member. | In my view, what I understand about quality health services is people should exactly receive that service that they are seeking. Also, prevention is an important part. Our responsibilities are preventive services too. Receiving only curative services is not a quality health service. |
| Population | View of community | Any mention of how leaders/staff view the community, perceptions of community members and patients, positive or negative |  |  | I think people trust this center very much. |

**Appendix table 3: Performance of best and worst performing primary health care centers in Province 1, Nepal^1^**

|  | Children under five with pneumonia who received antibiotics | Children under five years with diarrhea treated with zinc and ORS | Newborns with chlorhexidine ointment applied immediately after birth | Planned immunization clinics conducted | Planned immunization sessions conducted | Vaccine wastage rate | Overall performance score |
| --- | --- | --- | --- | --- | --- | --- | --- |
| **Best performers** | % (SD) | % (SD) | % (SD) | % (SD) | % (SD) | % (SD) | % (SD) |
| PHCC-1 | 100 (0) | 100 (0) | 100 (0) | 100 (0) | 100 (0) | 80 (5) | 97 (1) |
| PHCC-2 | 100 (0) | 100 (0) | 100 (0) | 100 (0) | 100 (0) | 77 (7) | 96 (2) |
| PHCC-3 | 100 (0) | 100 (0) | 100 (0) | 100 (0) | 100 (0) | 75 (6) | 96 (2) |
| PHCC-4 | 100 (0) | 92 (21) | 97 (10) | 100 (0) | 100 (0) | 82 (10) | 95 (5) |
| **Worst performers** | % (SD) | % (SD) | % (SD) | % (SD) | % (SD) | % (SD) | % (SD) |
| PHCC-5 | 20 (45) | 70 (29) | 96 (16) | 100 (0) | - | 60 (9) | 75 (14) |
| PHCC-6 | 20 (44) | 80 (28) | 99 (4) | 83 (39) | 83 (39) | 53 (18) | 75 (15) |
| PHCC-7 | 92 (17) | 64 (32) | 100 (0) | 91 (30) | 100 (0) | 51 (11) | 74 (26) |
| PHCC-8 | 56 (50) | 58 (27) | 72 (35) | 57 (39) | 92 (29) | 85 (16) | 70 (16) |

^1^Performance scores are determined as the average of six quality measures: 1) percent of children under five years with pneumonia who received antibiotics, 2) percent of children under five years with diarrhea treated with zinc and ORS, 3) percent of newborns who had chlorhexidine ointment applied immediately after birth, 4) percent of planned immunization clinics conducted, 5) percent of planned immunization sessions conducted, and 6) the vaccine wastage rate for BCG, Measles, DPT-HepB-Hib, Td, JE, Polio, PCV vaccines. Indicators were averaged for the month and then for the year for each facility. PHCC-5 did not report the percent of planned immunization sessions conducted so the indicator was excluded for this facility.

**Appendix figure 1: Performance scores of best and worst performing primary health care centers in Province 1, Nepal^1^**

^1^Performance scores are determined as the average of six quality measures: 1) percent of children under five years with pneumonia who received antibiotics, 2) percent of children under five years with diarrhea treated with zinc and ORS, 3) percent of newborns who had chlorhexidine ointment applied immediately after birth, 4) percent of planned immunization clinics conducted, 5) percent of planned immunization sessions conducted, and 6) the vaccine wastage rate for BCG, Measles, DPT-HepB-Hib, Td, JE, Polio, PCV vaccines.
